# Adherence to Guidelines for Diagnosis, Staging, and Treatment for Gastric Cancer in Italy According to the View of Surgeons and Patients

**DOI:** 10.3390/jcm13144240

**Published:** 2024-07-20

**Authors:** Manrica Fabbi, Marika Sharmayne Milani, Simone Giacopuzzi, Carlo De Werra, Franco Roviello, Claudia Santangelo, Federica Galli, Angelo Benevento, Stefano Rausei

**Affiliations:** 1 Department of General Surgery, Cittiglio-Angera Hospital, ASST Settelaghi, 21033 Varese, Italy; marikasharmayne.milani@asst-settelaghi.it (M.S.M.); stefano.rausei@asst-settelaghi.it (S.R.); 2 General and Upper GI Surgery Division, Department of Surgery, University of Verona, 37134 Verona, Italy; simone.giacopuzzi@univr.it; 3 Department of Advanced Biomedical Sciences, Federico II University Hospital, 80131 Naples, Italy; dewerra@unina.it; 4 Department of Medical Surgical Sciences and Neurosciences, Section of General Surgery and Surgical Oncology, Istituto Toscano Tumori (ITT), University Hospital of Siena, University of Siena, 53100 Siena, Italy; franco.roviello@gmail.com; 5 President of Association “Vivere Senza Stomaco”; claudia.santangelo@viveresenzastomaco.org; 6 Department of General Surgery, Gallarate Hospital, ASST Valle Olona, 21013 Gallarate, Italy; federica.galli@asst-valleolona.it (F.G.); angelo.benevento@asst-valleolona.it (A.B.)

**Keywords:** gastric cancer, gastrectomy, lymphadenectomy, guidelines, survey

## Abstract

**Background**: Despite the strong declining trends in incidence and mortality over the last decades, gastric cancer (GC) is still burdened with high mortality, even in high-income countries. To improve GC prognosis, several guidelines have been increasingly published with indications about the most appropriate GC management. The Italian Society of Digestive System Pathology (SIPAD) and Gastric Cancer Italian Research Group (GIRCG) designed a survey for both surgeons and patients with the purpose of evaluating the degree of application and adherence to guidelines in GC management in Italy. **Materials and Methods**: Between January and May 2022, a questionnaire has been administered to a sample of Italian surgeons and, in a simplified version, to members of the Patient Association “Vivere Senza Stomaco” (patients surgically treated for GC between 2008 and 2021) to investigate the diagnosis, staging, and treatment issues. **Results**: The survey has been completed by 125 surgeons and 125 patients. Abdominal CT with gastric hydro-distension before treatment was not widespread in both groups (47% and 42%, respectively). The rate of surgeons stating that they do not usually perform minimally invasive gastrectomy was 15%, but the rate of patients who underwent a minimally invasive approach was 22% (between 2011 and 2022). The percentage of surgeons declaring to perform extended lymphadenectomy (>D2) was 97%, although a limited lymph node dissection rate was observed in about 35% of patients. **Conclusions**: This survey shows several important discrepancies from surgical attitudes declared by surgeons and real data derived from the reports available to the patients, suggesting heterogeneous management in clinical practice and, thus, a not rigorous adherence to the guidelines.

## 1. Introduction

Gastric cancer (GC) is one of the most common malignant tumors worldwide. The incidence of GC varies widely depending on the region of the world, with the highest rates found in Eastern Asia (particularly in China, Korea, and Japan) and South America. In Western Europe, the incidence is highest in Southern and Eastern European countries and lowest in the Northern ones [1,2].

Despite the strong declining trends in incidence and mortality over the last decades, GC is still burdened with high mortality, even in high-income countries [3,4]. Hence, the continuous interest in improving GC management has led to the development of several guidelines in the last decade [5,6,7,8]. The guidelines emphasized the importance of a multidisciplinary team to discuss the correct conduct of GC patients, from preoperative staging to follow-up, as well as the indications to treat GC, especially in experienced, high-volume centers [5]. Studies have shown that treatment of GC in a high-volume center may result in better outcomes compared to low-volume centers [9,10], especially if expertise and adherence to guidelines support clinical practice. 

Despite the promising results emerging from multiple studies (either prospective or randomized trials) about GC survival, the improvement in clinical outcomes for this disease has been negligible in the last decades. The reasons for this phenomenon have yet to be clarified, although the quality of surgery and clinical management performed on controlled trials in contrast to real-world settings is one of the most plausible. Thus, ameliorating the adherence to guidelines and compliance with operative standards may be a way to improve outcomes in this aggressive disease. Nevertheless, information about the real degree of adherence to guidelines in gastric cancer is scarce [11,12] or completely absent in Italy.

This study reported, for the first time in GC clinical research, the outcomes of a survey specifically designed for both patients and surgeons by the Italian Society of Digestive System Pathology (SIPAD) and Gastric Cancer Italian Research Group (GIRCG) with the aim of evaluating the adherence to National and International Guidelines for GC in Italy [5,6,7,8,13,14].

## 2. Materials and Methods

SIPAD and GIRCG designed a survey about GC issues to be administered to two different groups. The first was represented by a sample of Italian surgeons operating either in high- or low-volume GC centers, belonging to both scientific society and their network; the second consisted of members of the Patient Association “Vivere Senza Stomaco”, including patients surgically treated in Italy for GC between 2008 and 2021, enrolled by voluntary application. The questionnaire was created using Google Forms and sent by e-mail between January and May 2022. A web-based survey, consisting of 29 questions, was developed by surgeons who have received advanced training in upper-gastrointestinal surgery. The questions investigate the diagnosis, staging, and treatment issues of GC. The version proposed to patients, also consisting of 29 questions, was simplified to improve understanding (Table 1a,b).

Considering the importance of lymphadenectomy, a specific question about factors affecting its extent was included in the surgeon’s questionnaire.

The final total of comparable item questions between the two groups was 10, and both survey questionnaires are shown in Table 2. The other questions were discarded from the final analyses because the answers provided by the patients’ group were incomplete and, therefore, the resulting data were not reliable for comparison. Patients were asked to complete the questionnaire using their discharge letter and pathological exam, with the help of their family physician. In the case of the absence of the type of lymphadenectomy performed on the discharge letter, patients were asked to write the number of lymph nodes removed declared on the histological examination report; the extended (D2) lymphadenectomy has been considered in cases of the removal of 25 nodes or more (according to Siewert’s principles) [15].

Table 3 shows the indications of the main GC guidelines in reference to the items compared between the two groups of this study. The data collected in the electronic survey application was transferred into a database created for this purpose and checked for completeness. Total or subtotal gastrectomies performed for GC from 2008 to 2021 were considered inclusion criteria, while the emergency surgery was excluded as well lack of declaration of type of lymphadenectomy performed or number of lymphonode removed. This study complied with the Declaration of Helsinki and was conducted in accordance with good clinical practice guidelines. Informed consent was obtained from all subjects involved in this study, and written informed consent has been obtained from the patients to publish this paper. 

The endpoint of this study was to evaluate the adherence to the guidelines in clinical practice by comparing the responses of the two groups.

## 3. Statistical Analysis

Outcomes were expressed as frequencies and percentages. Statistical analysis of these outcomes and their variation among surgeons and patients intergroup were performed using Pearson’s chi-square test and Fisher’s exact test.

In the case of ranked variables, a linear-by-linear association was used. A *p* value < 0.05 was considered statistically significant. Statistical analyses were performed using R tools. 

## 4. Results

In total, 136 patients and 125 surgeons answered the survey. In the first group, 11 patients were excluded since 5 underwent emergency gastric surgery, while in 6 patients, the type of lymphadenectomy performed or the number of lymph nodes removed was not specified. Baseline characteristics of the two groups are reported in Table 4 and Table 5, respectively. An almost homogeneous distribution of surgeons and patients originating from the various Italian regions was observed. Among patients, 33% decided to be operated on at a hospital outside their region of origin, of which 30 (73%) moved from Central-Southern to Northern Italy. The answers to comparable items given by the two groups to the questionnaire are reported in Table 6.

Regarding the clinical stage (cTNM) of the primary tumor, in both groups, the abdominal computed tomography (CT) scan was used more frequently without gastric hydro-distension (*p* = 0.44). Instead, the use of staging laparoscopy (SL) to complete clinical staging in cases of locally advanced GC (LAGC) was inconsistent between the two groups, since only 30% of patients with LAGC underwent SL compared to the 86% of surgeons declaring to always perform SL in LAGC (*p* < 0.05).

Another significant difference emerged about the surgical approach: 85% of surgeons declared to adopt minimally invasive (laparoscopic and/or robotic) surgery (MIS), but 79% of patients were treated with open surgery (*p* < 0.05). Actually, among the surgeons declaring to use MIS approaches, 42% delimit this application to only distal cancers and 8% to early tumors. However, although in the patient group, 31% and 21% had distal and early cancer, respectively, only a small percentage of them were treated with MIS (6 and 8 patients, respectively). Moreover, the patients rarely underwent an open total gastrectomy. In light of such a significant discrepancy between the two groups and considering the increasing diffusion of MIS in the last decade, the comparison was restricted to a more recent interval; however, the discrepancy among the two groups was still significant, even restricting cases from 2011 and 2019 (*p* < 0.05).

Significant differences also appear about the type of gastrectomy performed in cases of middle or lower-third gastric cancer: 95% of surgeons prefer a subtotal gastrectomy, whereas 67% of patients underwent total gastrectomy in these cases (*p* < 0.05).

The response about the type of lymphadenectomy also showed differences among the two groups, since 97% of surgeons declared to perform at least a D2 lymphadenectomy (in some cases, also D2 plus PAND lymphadenectomy), whereas about 35% of patients underwent a D1 or D1 plus lymphadenectomy (*p* < 0.05). This discrepancy remains significant, considering only patients from 2011 and 2019 (*p* < 0.05). With this specific regard, the surgeons were also interviewed on which factors would affect the extension of the lymphadenectomy: tumor staging, patients’ characteristics (age, comorbidity, and frailty), and surgical skills emerged among the most important factors (Table 7). 

No significant differences between the two groups emerge about the type of reconstruction performed after gastric surgery (with a clear majority favoring Roux-en-Y rather than Billroth II reconstruction), the use of a naso-gastric tube (NGT) after gastrectomy, and the employment of an abdominal drain (prevalently used and independently of the type of gastric resection). 

## 5. Discussion

Despite relevant improvements, GC still maintains a severe prognosis, sustaining active research and a continuous update of guidelines to optimize its management. Nevertheless, the extent of guidelines adoption, adherence, and compliance across surgeons and centers is poorly documented, leaving it unclear whether the scientific advance is followed by daily clinical application. 

In this study, a survey was conducted to compare the answers of surgeons and patients, with the aim of providing an overview of the application of guidelines during clinical practice, identifying potential bottlenecks and critical elements in their compliance or adherence, and evaluating their role in GC prognosis.

A first element emerging from the survey concerns the volume of centers and surgeons’ experiences: the vast majority of surveyed patients and surgeons derive from high-volume centers (>500 bed hospitals), although more than 40% of surgeons declared to manage less than 20 cases per year. The management of GC patients in specialized, high-volume centers would seem to be a major factor in improving their prognosis [8]. Dedicated centers should ensure appropriate expertise, including staff, materials, infrastructure, knowledge, and ongoing research, and are associated with improved surgical outcomes, significantly reducing the risk of perioperative morbidity and mortality [16,17,18,19]. However, even if high-volume centers do not necessarily mean high-volume surgeons, unfortunately, this real-world evidence cannot find improvements without a formal policy of centralization, as also emerged from this survey: 41% of patients have been recommended to move to specialized centers, even in other regions. Indeed, a mismatch was found between the number of gastrectomies performed in each Italian province and the real number of GCs diagnosed in the same zone, with a south-to-north trend displacement of patients, as noted also by Lorenzon et al., 2022 [10].

Our survey reveals an infrequent use of CT scans with gastric hydro-distension for GC staging, as confirmed by either surgeons or patients’ groups. According to both Italian and international guidelines [5,13], intravenous contrast-enhanced abdominal CT scans should be implemented with hydro-distension of the stomach to improve the accuracy of preoperative staging. Stomach CT with water-distension is less hindered by artifacts caused by air in the lumen, well showing detailed mucosal enhancement of cancer foci [20].

In clinical staging, staging laparoscopy (SL) should be performed in stage IB-III, according to current international guidelines [5,8,13], to exclude radiologically occult abdominal metastatic disease (in particular, peritoneal ones) in potentially resectable GC. SL sensitivity and specificity range between 65–95% and 80–100%, respectively, with an accuracy of up to 93% [21,22]. Moreover, the use of SL seems to prevent up to 20% of patients from undergoing unnecessary surgery and/or neoadjuvant therapy [21,22,23]. An unexpected outcome of the survey concerns the adoption of SL: 86% of surgeons claim to perform SL in LAGC, but only 30% of LAGC patients underwent SL. The reason for such a discrepancy is difficult to explain: assuming from an ethical point of view a perfect match between surgeons’ answers and their daily clinical work, the results can be interpreted as an effective deviation from guidelines compliance by a non-negligible part of Italian surgeons. However, the extent to which this putative neglection affects the GC prognosis remains to be established.

A significant discrepancy also emerges about the surgical approach: most surgeons declare to use MIS, while most of the patients underwent open surgery. In particular, almost half of the surgeons stated that they would limit MIS only for distal gastrectomy, preferring an open approach for total gastrectomy. However, only 19% of patients underwent distal gastrectomy performed by MIS. This divergence persists also considering only surgery performed after 2019, despite the publication of the KLASS-02 trial showing the benefits of MIS versus an open approach in terms of a lower complication rate, faster recovery, and less pain in LAGC [24]. Before KLASS-02, several randomized controlled trials demonstrated the advantages of laparoscopic gastrectomy compared to open distal gastrectomy in terms of lesser blood loss, faster time of bowel canalization and oral intake, earlier mobilization, reduced hospital stay, and post-operative complications. They also reported comparable overall and disease-specific survival rates for stage I gastric cancer [25,26], but also for LAGC, and comparable survival rates [24,27,28,29,30,31,32]. Although the feasibility, safety, and benefits of MIS for distal gastric cancer have been reported and widely accepted, its diffusion is still limited, especially in Western countries [33]. The data emerging from our survey seems to reflect the preference of Italian surgeons to adopt an open approach. An explanation for the distrust of using MIS seems to be related to the technical difficulty of its execution [34], since laparoscopic distal gastrectomy (LDG) is still regarded as a complex procedure, requiring training to achieve optimal results [35,36,37,38]. Such a finding would have been more expected for laparoscopic total gastrectomy (LTG). In fact, there is no consensus about the safety of LTG [7,39,40,41,42,43,44,45], and currently, Western guidelines do not include LTG as a standard treatment option [5,6]. Another plausible explanation is related to the ‘real-life’ conditions experimented by surgeons, often typically characterized by elderly patients with significant co-morbidities and advanced tumor stages, often treated with neoadjuvant therapy, and not always eligible for minimally invasive surgery [46,47]

According to the results of this survey, total gastrectomy seems to be the preferred procedure even in cases of middle or lower-third GC. Although almost all surgeons (95%) declare to perform distal gastrectomy, 67% of patients underwent total gastrectomy. Thus, apparently, a significant part of Italian surgeons perform total gastrectomy even for middle or lower-third gastric cancers, despite a negative proximal resection margin greater than 5 cm. The literature considers distal gastrectomy the gold standard procedure, either in early or locally advanced stages, improving short-term outcomes and providing comparable long-term prognosis under the precondition of a safe negative proximal resection margin [5,7,8,48].

An important aspect of the survey concerns the lymphadenectomy in the case of LAGC. Even if almost all surgeons declare to perform at least a D2 lymphadenectomy (97%), still a relevant rate of patients underwent insufficient lymph node (LN) dissection (about 35%). Even if the level of lymphadenectomy is not easily defined by patients, indirectly, the objective number of retrieved nodes in the pathological report is considered strongly related to the extension of nodal dissection. Hence, it is well known that there is a strict correlation between the number of LNs harvested and survival rate [49,50], with their metastasis being the most important prognostic factor in GC [51,52,53,54]. While the optimal number of retrieved LNs remains controversial, apparently, 16 LNs seems like the minimum threshold for a proper N staging, but the extended lymphadenectomy, statistically with more than 25 removed nodes according to Siewert’s principles [15], is the gold standard in LAGC, leaving a D1 or D1-plus only in carefully selected cases (high-risk patients or cT1N0 tumors not treatable by endoscopic resections) [5,7,8,14,55,56,57,58]. Insufficient LN removal emerged as a relevant outcome from the survey. Consistently with the results abovementioned, it is clear that patients’ age [59,60], BMI [61], and/or tumor stage [59,60,62] contribute to LN yield, as some patients are not suitable for D2 lymph node dissection, as well as adequate surgical skills. While patient and tumor-related factors are unmodifiable random factors, surgical skills are operator-dependent and, thus, variable from expert surgeons to those with an incomplete GC learning curve. Indeed, the literature recognizes a surgeon’s skill as a primary determinant of an optimal dissection. Also, the adoption of a different approach (MIS versus open) should have no impact on LNs harvest [63,64,65,66,67,68], in contrast to our survey, where the performance of some surgeons appears to be still affected by the surgical approach. 

Despite the lack of specific evident recommendations in clinical practice, the overlap between surgeons and patients’ responses about the type of reconstruction performed for restoring intestinal continuity after gastrectomy [5,69,70,71] and the use of prophylactic naso-gastric tubes [6,72,73] and abdominal drains [6,74] paradoxically represents a measure of the reliability of this analysis. 

Unless there is a significant difference between surgeons and patients, the survey underlines a tendency toward an early start of post-operative oral water intake, whose feasibility, safety, and benefits are extensively demonstrated [75,76]. Instead, discordance was observed for nutritionist involvement after discharge, since most patients were followed only in rare cases of dietary problems, while surgeons declared a more systematic involvement. The literature guidelines recommend an adequate follow-up after gastrectomy due to the patient’s weight loss and nutritional deficiencies [77,78] as well as malnutrition, directly correlated to worse short and long-term outcomes [79].

### Limitations of This Study

Apart from the relatively small cohort of surgeons and patients’ participants in the survey, a major limitation of this study was the recruitment of surgeons and patients. About surgeons, they were random but mostly occurring within the context of main scientific societies and, thus, probably not completely representative of the Italian gastric surgeons, as clearly emerged from the comparison with patients’ outcomes. On the other hand, the involvement of surgeons outside of scientific societies is extremely difficult since they are often disinclined to collaboration and research. Recruiting of patients is even more problematic since they have low affinity for scientific purview and often reject research matters; therefore, choosing to interview members of a Patient Association enrolled by voluntary application guarantees a more adequate question’s understanding as well as a greater adequacy of the answers with the therapy received, with also the help of their family physician in compiling the questionnaire.

Another limitation is that the random choice of patients and surgeons group prevents a direct comparison of answers and, thus, the evaluation of possible discrepancies between knowledge of the guidelines and real clinical practice. 

Moreover, centralization of GC cases to high-volume centers or high-volume surgeons is not the gold standard in Italy. According to the last edition of PNE (“Piano Nazionale Esiti”, 2023), just 12% of the GC patients have been managed in hospitals, with >20 GC cases per year. This wide distribution does not allow for accurate data. 

Another limitation is the inclusion of patients managed before the publication of the latest considered guidelines. However, considering that most important items have been minimally refined over years, this limitation does not affect the overall conclusions of this study. 

The inability to check the accuracy of information from patients is another factor to consider. To circumvent this problem, the formulation of questions was simplified for patients, and the questionnaire was completed with the help of their family physician.

Therefore, this analysis cannot presume to declare a general carelessness by surgeons in the management of GC, but it aims to give a warning about some potential significant deviations of clinical (and surgical) practice from guidelines.

## 6. Conclusions

This study reported the first survey in the literature to explore the real-world quality of surgery and clinical management in gastric cancer in Italy. The survey highlights remarkable discrepancies between the surgical attitudes declared by surgeons and the information reported by patients, suggesting heterogeneous management in clinical practice.

## Figures and Tables

**Table 1:** (**a**) Surgeons questionnaires. (**b**) Patients questionnaires.

(a)
**What type of medical specialty do you have?**
General surgery
Gastrointestinal surgery
Emergency surgery
**How old are you?**
<40 years old
>40 < 60 years old
>60 years old
**What type of hospital do you work at?**
University hospital
Non-university hospital
**How many hospital beds are there in the hospital where you work?**
<200 hospital beds
>200 <500 hospital beds
>500 hospital beds
**How many cases of gastric cancer do you manage annually?**
<10
>10 < 20
>20 < 50
>50
**Are you part of the MDTM?**
Yes, I am a member of MDTM
No, I am not a member of MDTM
There isn’t a MDTM in the hospital where I work
**Do you discuss gastric cancer patients at MDTM?**
Always
Never
Only in controversial management
There isn’t a MTDM in the hospital where I work
**If you are part of a MDTM, when do you usually discuss GC patients? **
Only after the completion of clinical staging
After completion of clinical staging and surgery
**For the histological typing on the endoscopic biopsy of the tumor before treatment, what type of diagnosis do you consider decisive (and do you repeat the examination if it is not available)?**
Adenocarcinoma
Adenocarcinoma with histological features (grading, WHO, or Lauren classification)
Adenocarcinoma with histological features and biomolecular characterization
**Have you ever referred a patient for genetic counseling?**
Yes
No
**What type of CT scan do you require for abdominal clinical staging (cTNM)?**
Abdominal CT scan with intravenous contrast
Abdominal CT scan with intravenous contrast and gastric hydro-distension
**Do you require an ultrasound endoscopy for the clinical staging (cT) of the tumor?**
Always
Never
Only for EGC
Only for AGC
**Do you require whole-body PET-CT for detecting distant metastatic disease (cM)?**
Always
Never
Only in cases of suspected metastasis on previous exams
**In case of LAGC, do you usually perform SL before neoadjuvant chemotherapy?**
No
Yes
**If feasible, do you perform a mini-invasive approach (laparoscopic/robotic)?**
Always
Never
Only for EGC
Only for distant tumors
**Do you perform a mediastinal lymphadenectomy on an EGJ tumor?**
Never
Only in Siewert 1
Only in Siewert 1-2
Always (Siewert 1-2-3)
**In cases of middle or lower-third gastric cancer, what type of gastrectomy do you perform?**
Subtotal gastrectomy
Total gastrectomy
**In the case of EGC with signet ring cells, what type of lymphadenectomy do you perform?**
D1
D1 plus
D2
D2 plus PAND
**In the case of LAGC, what type of lymphadenectomy do you usually perform?**
D1/D1 plus
D2
D2 plus PAND
**In your experience, which factors would affect the type of lymphadenectomy? *Score for each factor using numbers from 0 (poor) to 5 (very good), allocating weights to show the importance of each of these factors.***
Tumor staging
Surgical skills
Patient age
Patient comorbidity
Patient frailty
Nutritional status
Surgical approach
Anatomic conditions
**Which type of reconstruction after gastric surgery do you prefer?**
Roux-en-Y reconstruction
Billroth II reconstruction
**How do you manage post-operative pain?**
Epidural analgesia
Tap block
Continuous subcutaneous infusion
Intravenous analgesia
**Do you usually insert NGT after a gastrectomy?**
No
Yes
**Do you routinely insert at least one abdominal drain?**
No
Yes
**When does the patient usually start oral water intake?**
I POD
From or after II POD
**When do you usually stop an intravenous infusion?**
I POD
II POD
From or after III POD
**Do you routinely require a post-operative anastomotic leak test?**
Never
Always
Only after a total gastrectomy
**Is a nutritionist involved in patient follow-up after discharge?**
No, never
Yes, always
Only for patients with difficulties in resuming regular nutrition by mouth
**In your experience, who manages the oncological follow-up?**
Always the surgeon
Always the oncologist
Always the family doctor
Depends on tumor staging and the treatment performed
**(b)**
**When did you undergo a gastrectomy?**
1996–2000
2001–2005
2006–2010
2011–2015
2016–2020
2021
**How old were you at the time of the surgery?**
<40 years old
>40 < 60 years old
>60 < 80 years old
>80 years old
**What are your Italian regions of origin?**
From northern Italy
From central Italy
From southern Italy
**In which Italian region did you undergo surgery?**
In Northern Italy
In Central Italy
In Southern Italy
**How many hospital beds are there in the hospital where you underwent surgery?**
<200 hospital beds
>200 <500 hospital beds
>500 hospital beds
**Which specialist did you initially consult after the GC diagnosis?**
Surgeon
Oncologist
Other
**Was your gastrectomy performed as an emergency surgery after your access to the emergency department?**
Yes
No
**After GC diagnosis, were you advised to continue treatment at a more specialized center?**
Yes
No
**Were you given a multidisciplinary discussion report (surgeon–oncologist–radiotherapist, etc.)?**
Yes
No
I don’t know
**Did you undergo an abdominal CT scan prior to surgery?**
Yes and I could not drink anything before the exam
Yes and I had to drink at least half a liter of water before the exam
**In addition to the esophagogastroscopy and CT scan, did you undergo other exams before surgery?**
Abdominal ultrasound
Abdominal MRI
Ultrasound endoscopy
Whole-body PET-CT
Nothing
**Did you undergo neoadjuvant therapy before surgery?**
Yes
No
**If you had received neoadjuvant chemotherapy, did you undergo SL before starting chemotherapy?**
No
Yes
**Which surgical technique was used to perform your gastrectomy?**
Open approach
Laparoscopic/robotic approach
**Where was the location of your GC?**
Cardia
Fundus
Body
Antrum
**If your tumor was located in the esophagus-stomach area, did the surgery also involve the chest?**
No
Yes
I don’t know
**If the tumor was in the medium or lower part of the stomach, how much stomach was removed?**
Part of the stomach
Entire stomach
**Do you know which type of anastomosis was performed to restore intestinal continuity after gastrectomy?**
Roux-en-Y reconstruction
Billroth II reconstruction
I don’t know
**From your histopathology report, how many lymph nodes were removed during the surgery? (Only for patients with LAGC)**
<16
≥16
**Referring to your histological examination, could you specify T?**
1
2
3
4
**Referring to your histological examination, could you specify N?**
1
2
3
4
**Did you have a distant metastasis diagnosis before or during surgery?**
No
Yes
I don’t know
**If you had a distant metastasis, where were they?**
Peritoneal metastasis
Liver metastasis
Distant lymph nodes
Other
**What other diseases were you suffering from at the time of the surgery?**
Heart diseases
Diabetes
Liver disease
Arterial hypertension
**During the post-operative period, did you have NGT?**
No
Yes
**During the post-operative period, after how many days did you start to drink water?**
I POD
From or after II POD
**During post-operative period, did you have at least one abdominal drain?**
No
Yes
**Have you been examined by a nutritionist after surgery?**
No
Yes, but I didn’t have nutritional problems
Yes because I had nutritional problems
**Who monitored you during your oncological follow-up?**
The surgeon
The oncologist
The family doctor

MDTM: Multidisciplinary Team Meeting; EGC: early gastric cancer; LAGC: locally advanced gastric cancer; SL: staging laparoscopy; EGJ: esophagogastric junction; NGT: nasogastric tube; POD: post-operative day; GC: gastric cancer.

**Table 2:** Final total of comparable item questions between two groups.

Surgeons	Patients
**What type of CT scan do you require for abdominal clinical staging (cTNM)?**	**Did you undergo an abdominal CT scan prior to surgery?**
Abdominal CT scan with intravenous contrast	Yes and I could not drink anything before the exam
Abdominal CT scan with intravenous contrast and gastric hydro-distension	Yes and I had to drink at least half a liter of water before the exam
**In case of LAGC, do you usually perform SL before neoadjuvant chemotherapy?**	**If you had received neoadjuvant chemotherapy, did you undergo SL before starting chemotherapy?**
No	No
Yes	Yes
**If feasible, do you perform a mini-invasive approach (laparoscopic/robotic)?**	**Which surgical technique was used to perform your gastrectomy?**
No	Open approach
Yes	Laparoscopic/robotic approach
**In cases of middle or lower-third gastric cancer, what type of gastrectomy do you perform?**	**If the tumor was in the medium or lower part of the stomach, how much stomach was removed?**
Subtotal gastrectomy	Part of the stomach
Total gastrectomy	Entire stomach
**In the case of LAGC, what type of lymphadenectomy do you usually perform?**	**From your histopathology report, how many lymph nodes were removed during the surgery? (Only for the patient with LAGC)**
D1/D1 plus	<16
≥D2	≥16
**Which type of reconstruction after gastric surgery do you prefer?**	**Do you know which type of anastomosis was performed to restore intestinal continuity after gastrectomy?**
Roux-en-Y reconstruction	Roux-en-Y reconstruction
Billroth II reconstruction	Billroth II reconstruction
**Do you usually insert NGT after gastrectomy?**	**During the post-operative period, did you have NGT?**
No	No
Yes	Yes
**Do you routinely insert at least one abdominal drain?**	**During the post-operative period, did you have at least one abdominal drain?**
No	No
Yes	Yes
**When does the patient usually start oral water intake?**	**During the post-operative period, after how many days did you start to drink water?**
I POD	I POD
From or after II POD	From or after II POD
**Is a nutritionist involved in patient follow-up after discharge?**	**Have you been examined by a nutritionist after surgery?**
No, never	No
Yes, always	Yes, but I didn’t have nutritional problems
Only for patients with difficulties resuming regular nutrition by mouth	Yes because I had nutritional problems

SL: staging laparoscopy; LAGC: locally advanced gastric cancer; NGT: nasogastric tube; POD: post-operative day.

**Table 3:** Indications of the main GC guidelines in reference to the items compared between two groups.

Questions	Guidelines
**Type of CT scan for abdominal clinical staging**	Abdominal CT scan with intravenous contrast and gastric hydro-distension [5,13].
**Staging laparoscopy in advanced gastric cancer**	Staging laparoscopy is recommended in all stage IB-III gastric cancers that are considered potentially resectable to exclude radiologically and macroscopically occult peritoneal metastatic disease [5,8,13,14].
**Surgical approach**	Laparoscopic gastric resection for GC is an option that should be considered in patients with EGC; laparoscopic surgery is feasible also for AGC, but solid data on the advantages and oncologic efficacy of this approach coming from randomized trials are lacking [5,6]. Trials from East Asia in early and advanced (T2–T4a) gastric cancer have shown that laparoscopic distal gastrectomy is non-inferior with regard to oncological outcomes, with improved short-term outcomes [8,14].
**Gastrectomy in middle or lower-third gastric cancer**	Distal gastrectomy should be preferred when an adequate proximal resection margin can be obtained for distal tumors [5,8,14].
**Lymphadenectomy**	The standard treatment for potentially curative resection is D2, even after neoadjuvant treatment [5,8,14]. Lymph node dissection for T1 tumors may be confined to perigastric lymph nodes and include local N2 nodes [8,14].
**Type of reconstruction after gastric surgery**	After distal gastrectomy, Roux-en-Y reconstruction seems superior to Billroth I and Billroth II reconstructions in terms of functional outcomes and long-term endoscopic results [5].
**Use of a nasogastric tube after a gastrectomy**	Nasogastric tubes should not be used routinely in the setting of enhanced recovery protocols in gastric surgery [6].
**Use of abdominal drain**	The use of abdominal drainage to reduce related complications and accelerate patient recovery is not recommended; however, the level of evidence is low. In particular, the use of drainage after total gastrectomy is still widely debated in the context of the ERAS programs [6].
**Timing to start oral water intake**	Early administration of oral liquids from the first post-operative day [6].
**Nutritionist involvement after discharge**	Follow-up should include lifetime monitoring of the nutritional sequelae of gastrectomy, including, but not limited to, adequate vitamin B12, iron, and calcium replacement [6].

**Table 4:** Surgeons group’s characteristics.

	Surgeons (125)
**Age**	
<40	48 (39%)
>40 < 60	54 (43%)
>60	23 (18%)
**Number of cases of gastric cancer managed annually**	
<10	11 (9%)
>10 < 20	37 (30%)
>20 < 50	54 (43%)
>50	23 (18%)
**Number of hospital beds in the hospital where surgeons work**	
<200 hospital beds	12 (10%)
>200 <500 hospital beds	40 (32%)
>500 hospital beds	73 (58%)

**Table 5:** Patients group’s characteristics.

Characteristics	Patients (125)
**Year of gastrectomy**	
2008–2010	7 (6%)
2011–2015	24 (19%)
2016–2020	67 (54%)
2021	27 (21%)
**Age at the time of surgery**	
<40	10 (8%)
>40 < 60	61 (49%)
>60 < 80	53 (42%)
>80	1 (1%)
**Origin of the patient**	
From northern Italy	42 (33%)
From central Italy	51 (41%)
From southern Italy	32 (26%)
**Number of patients who have chosen to be operated on in a hospital in a region other than that of their origin**	41 (33%)
**Number of hospital beds in the hospital where the patient underwent surgery**	82 *
<200 hospital beds	14 (17%)
>200 <500 hospital beds	19 (23%)
>500 hospital beds	49 (60%)
**Gastric cancer location**	
Cardia	31 (25%)
Fundus	22 (18%)
Body	33 (26%)
Antrum	39 (31%)
**Type of gastrectomy**	
Total gastrectomy	89 (71%)
Subtotal gastrectomy	36 (29%)

* Data are missing.

**Table 6:** Results.

Answers	Surgeons (125)	Patients (125)	*p* Value **
**Type of CT scan for abdominal clinical stadiation**	**125**	**125**	**0.44**
Abdominal CT scan	66 (53%)	73 (58%)	
Abdominal CT scan with gastric hydro-distension	59 (47%)	52 (42%)	
**Staging laparoscopy in advanced gastric cancer**	**125**	**53**	**<0.05**
No	17 (14%)	37 (70%)	
Yes	108 (86%)	16 (30%)	
**Surgical approach**	**125**	**125**	**<0.05**
Open	19 (15%)	99 (79%)	
Laparoscopic/robotic approach	106 (85%)	26 (21%)	
***Surgical approach (since 2011)***	**125 **	**118 **	**<0.05 **
*Open*	19 (15%)	92 (78%)	
*Laparoscopic/robotic approach*	106 (85%)	26 (22%)	
***Surgical approach (since 2019)***	**125 **	**57 **	**<0.05 **
*Open*	19 (15%)	40 (70%)	
*Laparoscopic/robotic approach*	106 (85%)	17 (30%)	
**Gastrectomy in middle or lower-third gastric cancer**	**125**	**72**	**<0.05**
Subtotal gastrectomy	119 (95%)	24 (33%)	
Total gastrectomy	6 (5%)	48 (67%)	
**Lymphadenectomy**	**125**	91 *	**<0.05**
D1/D1 plus	4 (3%)	32 (35%)	
≥D2	121 (97%)	59 (65%)	
***Lymphadenectomy (since 2011)***	**125**	**87**	**<0.05**
*D1/D1 plus*	4 (3%)	30 (34%)	
*≥* *D2*	121 (97%)	57 (66%)	
***Lymphadenectomy (since 2019)***	**125**	**45**	**<0.05**
*D1/D1 plus*	4 (3%)	13 (29%)	
*≥* *D2*	121 (97%)	32 (71%)	
**Type of reconstruction after gastric surgery**	**125**	**125**	**0.14**
Roux-en-Y reconstruction	97 (78%)	107 (86%)	
Billroth II reconstruction	28 (22%)	18 (14%)	
**Use of a nasogastric tube after a gastrectomy**	**125**	**125**	**0.98**
No	31 (25%)	30 (24%)	
Yes	94 (75%)	95 (76%)	
**Use of abdominal drains**	**125**	123 *	**0.64**
No	10 (8%)	7 (6%)	
Yes	115 (92%)	116 (94%)	
**Timing to start oral water intake**	**125**	113 *	**<0.05**
POD 1	31 (25%)	11 (10%)	
≥POD2	94 (75%)	102 (90%)	
**Nutritionist involvement after discharge**	**125**	**125**	**<0.05**
No	4 (3%)	44 (35%)	
Yes	76 (61%)	36 (29%)	
Only for patients with nutritional problems	45 (36%)	45 (36%)	

POD: post-operative day; * Data missing; ** *p*-value: Chi-square/Fisher exact tests.

**Table 7:** Factors influencing the type of lymphadenectomy.

	Factors Answers (n. surgeons)							
Score *	Tumor Staging	Surgical Skills	Patient Age	Patient Comorbidity	Patient Frailty	Nutritional Status	Surgical Approach	Anatomic Conditions
1	2.4% (3)	14.3% (18)	5.6% (7)	4.8% (6)	4% (5)	9.5% (12)	39.7% (50)	13.5% (17)
2	7.1% (9)	12.7% (16)	8.7% (11)	12.7% (16)	11.1% (14)	14.3% (18)	17.5% (22)	23.8% (30)
3	22.2% (28)	18.3% (23)	29.4% (37)	31% (39)	31% (39)	37.3% (47)	25.4% (32)	38.1% (48)
4	29.4% (37)	28.6% (36)	34.9% (44)	28.6% (36)	27.8% (35)	26.2% (33)	10.3% (13)	17.5% (22)
5	38.9% (49)	26.2% (33)	21.4% (27)	23% (29)	26.2% (33)	12.7% (16)	7.1% (9)	7.1% (9)

* Score for each factor using numbers from 0 (not important) to 5 (very important) and allocating weights to show the importance of each of these factors.

## Data Availability

Data supporting the findings of this study are available from the corresponding author upon request.

## Supplementary Materials

The following supporting information can be downloaded at: https://www.mdpi.com/article/10.3390/jcm13144240/s1.
