# Supplementary material for: Adherence to Guidelines for Diagnosis, Staging, and Treatment for Gastric Cancer in Italy According to the View of Surgeons and Patients
Source: J Clin Med. 2024 Jul 20;13(14):4240. doi: 10.3390/jcm13144240 (PMC11277783; doi:10.3390/jcm13144240)
Supplement: Supplementary file 1 [file jcm-13-04240-s001.zip › jcm-3099715-supplementary.pdf]

SUPPLEMENTAL MATERIAL

List of Italian surgeons that answered the survey.

First Name	Last name	Affiliation
Vittorio	Albino	Division of Abdominal Surgical Oncology, Hepatobiliary Unit, Istituto Nazionale Tumori, IRCCS - Fondazione G Pascale, Napoli, Italy
Mariella	Alloggio	Upper G.I. Surgery Division, University of Verona, Verona, Italy
Maria Raffaella	Ambrosio	Pathology Department, "Azienda USL, Tuscany North-West", Pisa, Italy
Gabriele	Anania	Department of Medical Science, University of Ferrara, Ferrara, Italy
Jacopo	Andreuccetti	Department of General Surgery 2^, ASST Spedali Civili di Brescia, Brescia, Italy
Maria Costanza	Aquilano	Institute of Pathology ASST Niguarda, Milano, Italy
Gabriela Aracelly	Arroyo Murillo	Department of general surgery, ULSS3 Serenissima, Mestre, Italy
Paolo	Aurello	Department of Medical and Surgical Sciences and Translational Medicine, Sapienza University of Rome, Sant'Andrea Hospital, Roma, Italy
Francesco Giuseppe Aldo	Badessi	Department of General Surgery, "Clinica Sant'Elena" - Quartu Sant'Elena, Italy
Gianluca	Baiocchi	Surgical Clinic, Department of Experimental and Clinical Sciences, University of Brescia, Brescia, Italy
Gianandrea	Baldazzi	Department of Surgery, ASST Ovest Milanese, Legnano Hospital, Legnano, Italy
Serena	Battista	Pathology Unit, Azienda Sanitaria Universitaria Friuli Centrale S. Maria della Misericordia Hospital, Udine, Italy
Claudio	Belluco	Surgical Oncology, Department of Surgery, Centro di Riferimento Oncologico di Aviano (CRO), IRCCS, Aviano, Italy
Lapo	Bencini	Division of Oncologic Surgery and Robotics, Department of Oncology, Careggi University Hospital, Firenze, Italy
Mattia	Berselli	General Surgery Department, Varese Hospital, ASST Sette Laghi, Varese, Italy
Alberto	Biondi	General Surgery Unit, Fondazione Policlinico Universitario A Gemelli IRCCS, Roma, Italy
Antonio	Biondi	Department of General Surgery and Medical-Surgical Specialties, University of Catania, Catania, Italy
Giuseppe	Boccia	Department of Public Health, University of Naples Federico II, Napoli, Italy
Selene	Bogoni	Department of General Surgery, Poliambulanza Foundation Hospital, Brescia, Italy
Andrea	Brandimarte	Department of General Surgery, Azienda Ospedaliera Carlo Poma, Mantova, Italy

Federico	Cammillini	Department of Medicine, Surgery and Neurosciences, Unit of General Surgery and Surgical Oncology, University of Siena, Siena, Italy
Ludovico	Carbone	Department of Medicine, Surgery and Neurosciences, University of Siena, Siena, Italy
Francesca	Cardella	Surgical Oncology of Gastrointestinal Tract Unit, Vanvitelli University, Napoli, Italy
Marco	Caricato	Department of Colorectal Surgery, Colorectal Surgery Unit, Campus Bio-Medico University, Roma, Italy
Chiara	Cascone	Department of Surgery, University Campus Bio-Medico di Roma, Roma, Italy
Francesco	Casella	Upper GI Surgery Division, University of Verona, Verona, Italy
Paolo	Cerri	Department of Surgery, "Val Vibrata - Sant'Omero", Teramo, Italy
Damiano	Chiari	Department of General Surgery, Istituto Clinico Humanitas Mater Domini, Castellanza, Varese, Italy
Chiara	Cipollari	Department of General Surgery, ULSS 6-Euganea, Cittadella, Italy
Alessandro	Coppola	Department of Surgery, Sapienza Università di Roma, Roma, Italy
Italo	Corsale	General Surgery Unit, SS. Cosma e Damiano Hospital, Pescia, Italy
Maria Laura	Cossu	General Surgery Unit 2 "Clinica Chirurgica" Medical, Surgical and Experimental Sciences Department, University of Sassari, Sassari, Italy
Antonio	Costanzo	Humanitas Clinical and Research Center, Scientific Institute for Research, Hospitalization and Healthcare, Rozzano, Italy
Luigi	Cristadoro	General Surgery, "C. Poma" Hospital, Pieve di Coriano, Mantova, Italy
Anna	D’Amore	Department of Clinical Medicine and Surgery, "Federico II" University of Naples, Napoli, Italy
Domenico	D’Ugo	Department of Surgery, Fondazione Policlinico Universitario Agostino Gemelli, Roma, Italy
Giorgio	Dalmonte	Unit of General Surgery, Parma University Hospital, University of Parma, Parma, Italy
Stefano	De Pascale	Digestive Surgery, European Institute of Oncology IRCCS, Milano, Italy
Nicola	De Ruvo	Department of Surgery, University of Modena and Reggio Emilia, Policlinico of Modena, Modena, Italy
Matteo	Desio	Varese General Surgery, Department of Surgery, ASST SetteLaghi, Italy
Natale	Di Martino	Department of Surgery, University of Campania "L. Vanvitelli", Napoli, Italy
Ugo	Elmore	Department of Gastrointestinal Surgery, San Raffaele Scientific Institute, Milan, Italy

Francesco	Favi	General, Emergency and Trauma Surgery Department, Bufalini Hospital, Cesena, Italy
Uberto Romario	Fumagalli	Digestive Surgery, European Institute of Oncology IRCCS, Milano, Italy
Luigi	Funicelli	Department of Radiology, European Institute of Oncology IRCCS, Milano, Italy
Gennaro	Galizia	Division of GI Tract Surgical Oncology, Department of Surgical Sciences, University of study of Campania "Luigi Vanvitelli", Napoli, Italy
Raffaele	Galleano	General Surgery, Santa Corona Hospital, Pietra Ligure, Italy
Giovanni	Gambino	General Surgery, ASP of Trapani, Trapani, Italy
Roberta	Gelmini	Department of Surgery, University of Modena and Reggio Emilia, Policlinico of Modena, Modena, Italy.
Irene	Gentile	Department of General Surgery, Sacred Heart - Don Calabria Hospital, Negrar, Verona, Italy
Paola	Germani	Division of General Surgery, Department of Medical and Surgical Sciences, ASUGI, Trieste, Italy
Michele	Ghidini	Medical Oncology Unit, Fondazione IRCCS Ca' Granda Ospedale Maggiore Policlinico, Milano, Italy
Elson	Gjoni	General Surgery Unit, Vimercate Hospital, ASST Vimercate, ASST della Brianza, Vimercate, Italy
Vittoria	Grammatico	General Surgery, ASL TO5, Carmagnola, Italy
Luigina	Graziosi	General and Emergency Surgical Department, S. Maria della Misericordia Hospital and University, Perugia, Italy
Monica	Gualtierotti	Division of Minimally-Invasive Surgical Oncology, Niguarda Cancer Center, ASST Grande Ospedale Metropolitano Niguarda, Piazza dell'Ospedale Maggiore, Milano, Italy
Maddalena	Leongito	Gastro-pancreatic Surgery Department, Istituto Nazionale per lo Studio e la Cura dei Tumori "Fondazione Giovanni Pascale" IRCCS, Napoli, Italy
Federica	Maffeis	General Surgery, S. Maria degli Angeli Hospital, Pordenone, Italy
Alessandra	Marano	Department of Chemical Sciences, University of Naples "Federico II", Napoli, Italy
Mario	Martinotti	Division of Surgery, Cremona Hospital, Cremona, Italy
Martina	Martorana	U.O. Oncology Hospital, A.R.N.A.S. Ospedali Civico Di Cristina Benfratelli, Palermo, Italy
Gennaro	Mazzarella	Department of General and Emergency Surgery, San Filippo Neri Hospital, Roma, Italy
Irene	Melfa	General Surgery, AOUP Paolo Giaccone di Palermo, Palermo, Italy
Marco	Milone	Department of General and Emergency Surgery, Azienda Ospedaliera Universitaria "Federico II," Napoli, Italy
Silvia	Ministrini	Department of Experimental and Clinical Sciences, University of Brescia, Brescia, Italy
Luigi	Monaco	Surgery Department, "Villa Esther" Hospital, Via Due Principati, Avellino, Italy

Paolo	Morgagni	General and Oncologic Surgery, "Morgagni-Pierantoni" Hospital, Forlì, Italy
Abdallah	Moukachar	Department of Surgery, ASST Ovest Milanese, Legnano Hospital, Legnano, Italy
Bruno	Nardo	Department of Pharmacy, Health and Nutritional Sciences, University of Calabria, Rende, Italy, General Surgery Unit, Department of Surgery, A.O. Annunziata, Cosenza, Italy
Stefano	Olmi	Surgeon of General and Oncologic Surgery Department, Centre of Advanced Laparoscopic Surgery, Centre of Bariatric Surgery, San Marco Hospital GSD, Zingonia, Italy
Elena	Orsenigo	Department of General and Emergency Surgery, IRCCS San Raffaele Scientific Institute, Milano, Italy
Davide	Papis	General Surgery Unit, Del Mare Hospital, Napoli, Italy
Corrado	Pedrazzani	Division of General and Hepatobiliary Surgery, Department of Surgical Sciences, Dentistry, Gynaecology and Paediatrics, University of Verona, Verona, Italy
Roberto	Peltrini	Department of Public Health, University of Naples Federico II, Napoli, Italy
Andrea	Peri	Department of Surgery, University of Pavia and Fondazione IRCCS Policlinico San Matteo, Pavia, Italy
Michele	Pisano	General Surgery I, ASST Papa Giovanni XXIII Hospital, Bergamo, Italy
Roberto	Polastri	Department of Maternal-Infant Medicine, University Hospital "Degli Infermi", Ponderano, Biella, Italy
Nazario	Portolani	Department of Clinical and Experimental Sciences, Surgical Clinic, University of Brescia, Brescia, Italy
Alessandro	Puzziello	General Surgery Unit, AOU San Giovanni di Dio e Ruggi d'Aragona, University of Salerno, Largo Città di Ippocrate, Salerno, Italy
Salvatore	Ramuscello	Surgical Department, Chioggia Hospital, Venezia, Italy
Valerio	Ranieri	General Surgery, Istituti Ospitalieri di Cremona, Cremona, Italy
Emanuele	Rausa	Division of Surgical Oncology, ASST Bergamo Ovest, Treviglio, Italy
Rossella	Reddavid	Department of Oncology, University of Torino, Orbassano, Italy
Fausto	Rosa	Digestive Surgery Unit, Fondazione Policlinico Universitario Agostino Gemelli IRCCS, 00168 Rome, Italy
Luca	Rossit	Department of General Surgery, Azienda Ospedaliero-Universitaria of Udine Santa Maria della Misericordia, Udine, Italy
Massimiliano	Salati	Oncology Unit, University Hospital of Modena, Modena Cancer Centre, Modena, Italy
Paolo	Sammartino	CRS and HIPEC Unit, Pietro Valdoni, Umberto I Policlinico di Roma, Roma, Italy
Antonio	Santangelo	Università degli Studi di Udine, Medical, Udine, Italy
Stefano	Scabini	IRCCS Ospedale Policlinico San Martino, Genoa, Italy

Marco	Scatizzi	Department of General Surgery and Surgical Specialties, Unit of General Surgery, Santa Maria Annunziata Hospital, Central Tuscany Local Health Company, Firenze, Italy
Maximilian	Scheiterle	Emergency Surgery, Careggi University Hospital, Firenze, Italy
Carlo	Socci	Transplant and Metabolic/Bariatric Surgery Unit, IRCCS Ospedale San Raffaele, Milano, Italy
Leonardo	Solaini	Department of Medical and Surgical Sciences-DIMEC, Alma Mater Studiorum, University of Bologna, Bologna
Lucio	Taglietti	Esine General Hospital, Department of Surgery, ASST Valcamonica, Breno, Italy
Giuseppe	Tirone	Department of General Surgery, S. Chiara Hospital, Trento, Italy
Beatrice	Torre	Policlinico Sant'Orsola Bologna U.O. Chirurgia Tratto Alimentare, Bologna, Italy
Elio	Treppiedi	Department of General Surgery, Fondazione Poliambulanza-Istituto Ospedaliero, Brescia, Italy
Roberta	Tutino	Department of Surgical Oncological and Stomatological Disciplines, Unit of General and Emergency Surgery, University of Palermo, Palermo, Italy
Fabio	Uggeri	Department of Surgery and Translational Medicine, Foundation IRCCS San Gerardo dei Tintori, University of Milano-Bicocca, Monza, Italy
Michele	Valmasoni	Department of Surgery, Oncology and Gastroenterology, 3rd Surgical Clinic, University of Padua, Padova, Italy
Augusto	Verzelli	General Surgery, Fabriano Hospital, Fabriano, Italy
Jacopo	Viganò	Unit of General Surgery I, Fondazione I.R.C.C.S. Policlinico San Matteo, Pavia, Italy
Paolo	Viganò	Department of Infectious Diseases, Western Milan Area Hospital Consortium, Legnano General Hospital, Milan, Italy
Antonio	Vitiello	Department of General and Emergency Surgery, Azienda Ospedaliera Universitaria "Federico II," Napoli, Italy
Andrea	Zanoni	General Surgery, Rovereto Hospital, Azienda Provinciale per i Servizi Sanitari of Trento, Rovereto, Trento, Italy
